# Protective effects of silymarin-loaded chitosan nanoparticles in the diet-induced hyperlipidemia rat model

**DOI:** 10.22038/IJBMS.2024.74490.16179

**Published:** 2024

**Authors:** Vahid Akheratdoost, Negar Panahi, Shahabeddin Safi, Faraz Mojab, Ghasem Akbari

**Affiliations:** 1 Department of Veterinary Basic Sciences, Science and Research Branch, Islamic Azad University, Tehran, Iran; 2 Department of Veterinary Pathobiology, Science and Research Branch, Islamic Azad University, Tehran, Iran; 3 Department of Pharmacognosy, School of Pharmacy, Shahid Beheshti University of Medical Sciences, Tehran, Iran; 4 Department of Veterinary Clinical Sciences, Science and Research Branch, Islamic Azad University, Tehran, Iran

**Keywords:** Antiobesity, Body mass index, Chitosan, Dyslipidemia, High cholesterol diet, HOMA-IR, Silymarin

## Abstract

**Objective(s):**

Obesity is a metabolic syndrome that leads to many chronic diseases worldwide. In this study, we investigate the antihyperlipidemic activities of chitosan nanoparticles (CH NPs) on silymarin (SIL) as a carrier in the drug delivery system that can improve some biochemical parameters and hormones in the model of hyperlipidemic rats receiving a high-fat diet (HFD).

**Materials and Methods:**

Physicochemical characterization of silymarin-loaded chitosannanoparticles (CH-SIL NPs) was done by Fourier-transform infrared (FTIR) spectroscopy, dynamic light scattering (DLS), and drug loading efficiency (LE). Diet-induced hyperlipidemic rats were treated with SIL (15 mg/kg/day) and CH-SIL NPs(15 mg/kg/day) for twelve weeks orally daily. The body weight loss (BW), food consumption, serum total cholesterol (TC), triglycerides (TG), high-density lipoprotein (HDL), levels of fasting blood glucose (FBG) in serum, serum insulin, cortisol, testosterone, and brain neuropeptide Y (NPY), Y1 and Y5 receptor mRNA expression were analyzed.

**Results:**

A significant reduction in BW and food consumption from 417 ± 16 g and 33 ± 1.03 in group HFD to 338 ± 10 g and 17.33 ± 1.02 in group CHS+HFD was observed, respectively. This data revealed that CH-SIL NPs improved hyperlipidemia, hyperinsulinemia, and hyperglycemia, reduced serum cortisol, and down-regulated NPY and Y1R with a significant increase in HDL and testosterone hormones compared to the control group.

**Conclusion:**

The developed Sil-loaded CH NPs were good agents for improving efficacy. It is the first report of the proposed weight loss mechanism of SIL CH NPs, thereby providing information about the anti-hyperlipidemic and antihyperglycemic effects of silymarin-loaded chitosan nanoparticles, a natural food with proper effects against metabolic disorders in case of hyperlipidemia that may lead to obesity and up-regulation of brain NPY.

## Introduction

Obesity is a threatening factor for developing immune-mediated and inflammatory-mediated diseases, including dyslipidemia, hypertension (1), insulin resistance, cancer, musculoskeletal disorders, and cardiovascular disease (2). It reduces immune response to infectious agents, especially during the COVID-19 pandemic (3). High plasma concentrations of total cholesterol (TC), low-density lipoprotein cholesterol (LDL-C), and triglycerides (TG), as well as a low level of high-density lipoprotein cholesterol (HDL-C), are the abnormal features in dyslipidemia (4). Currently, there is a developing consumption of functional foods, including proteins, polyphenols, dietary fiber, phytosterols, and long-chain polyunsaturated fatty acids (PUFAs), as capable co-adjuvants in the pharmacological treatment of dyslipidemia. The arcuate nucleus of the hypothalamus (ARC) is the most virtual central region in the central nervous system that regulates energy homeostasis, including NPY-expressing neurons to send hormonal and nutrient-related signals of appetite, feeding, and metabolism (5). NPY is considered a crucial biomarker of metabolic syndromes in obesity (2). Additional research exhibited that specific hypothalamus regions receive signals from peripheral hormones, including insulin, ghrelin, and leptin, which lead to food intake or satiety (6). Dysregulation of these hormonal signals could cause weight gain and insulin resistance and elevate the risk of other metabolic diseases (7). NPY mediates appetite-stimulating activities via various NPY G-protein coupled receptors (NPYRs), including Y1, Y2, Y4, Y5, and Y6. Among these, the anorexigenic signals modulated via Y1 and Y5 receptors (2, 8). Recent findings on NPY neurons targeted genes, particularly antagonists of Y1 and Y5 receptors, compounds, and drugs, must be identified as beneficial agents that can develop for treating obesity.

Polyphenols are critical in the obesity-related modulation of lipid metabolism. The flavonolignans in the *Silybum marianum* (silymarin) prevent cytotoxins from entering the cells, thus protecting the liver cells (9). Silymarin (SIL) has shown antihyperglycemic (10), anti-inflammatory (11), cardioprotective, anti-oxidant, and hepatoprotective effects (12). Since SIL has these functions and obesity was associated with hyperlipemia, hormonal disorder, and overexpression of brain NPY, we conducted studies on SIL against weight gain to search whether there was a fundamental mechanism between silymarin-loaded chitosan and the weight loss effect. 

The poor bioavailability of SIL is the crucial factor restraining its use. It depends on factors such as weak water solubility. For drugs with poor water solubility/bioavailability, fast disintegration is essential to obtain a fast drug absorption/effect (13).With the advent of site-specific drug delivery science for sustained and controlled release of drugs, a new pathway has been developed to enhance oral absorption and, at the same time, achieve high efficacies of medicines. Thus, many drugs have been investigated *in vitro *and* in vivo* with different carriers. Chitosan (CH), as the positive charge polysaccharide, has remarkable properties, including high bioavailability, super biodegradability, high biocompatibility, mucoadhesiveness, and nontoxicity. Additionally, this polymer can open the tight intercellular connections of epithelia to carry various hydrophilic and hydrophobic drugs (14, 15). According to the chemical structure of CH, an amine group is bonded to carbon 2 in glucose, making the compound a polycation in acidic environments and capable of revealing the mucoadhesive feature in the intestinal mucosa (16). A reduction in testosterone levels in HFD-induced obesity rats may be due to testosterone’s conversion into estrogen, reducing testicle stimulation or lipid metabolic disorder causing male reproductive function loss (17).

Therefore, lipid-lowering and NPY down-regulation pharmacological interventions might reduce the risk of hyperlipidemia-induced serum hormonal complications such as testosterone decline, hyperinsulinemia, and increased cortisol levels. Hence, this study aimed to evaluate the effect of silymarin-loaded chitosan on lipid profile, blood glucose, serum insulin, cortisol, testosterone, and brain neuropeptide Y(NPY), Y1, and Y5 receptor mRNA expression in hyperlipidemic male Wistar rats receiving a high-fat diet. 

## Materials and Methods

***Materials***

Chitosan (low molecular weight) and silymarin were purchased from Sigma Aldrich (Germany). Penta-sodium triphosphate (Merck KGaA, Germany) was employed as a cross-linker. Cholesterol powder was obtained from Nacalai Tesque (Kyoto, Japan). All other chemicals were of analytical grade.

***Preparation of silymarin-loaded chitosan nanoparticles***

The formulations of silymarin-loaded chitosan nanoparticles (CH-SIL NPs) (15) were prepared with approximate modifications (18). First, chitosan (CH, 1 g) was dissolved in 50 ml of glacial acetic acid 1% at 40 °C to make a clear solution for 40 min. Second, SIL (0.5 g) was added to the CH solution under stirring for 60 min (mixture A). Third, 0.2 g of sodium tripolyphosphate (TPP) was dissolved in 20 ml of deionized water and dropped into mixture A (1 ml/min) under vigorous mechanical stirring for 30 min at room temperature. Subsequently centrifugation at 13000 rpm for 15 min and washing two-wise with deionized water, the cross-linked CH-SIL NPs were collected and dried at 40 °C. Some dried nanoparticles were dispersed in deionized water during administration (19).

***Characterization of CH-SIL NPs***

*Fourier-transform infrared (FTIR) spectroscopy analysis*

The chemical structure and functional groups of Sil, CH, and Sil-loaded CH NPs were analyzed by infrared spectroscopy (Tensor II, Bruker, Germany) using the KBr disk method.

*Zeta potential and particle size*

The surface charge (zeta potential), size distribution, polydispersity index (PDI), and mean size of the SIL-loaded CH NPs were assessed by Nano-ZS SZ- 100 (Horiba, Japan) using the dynamic light scattering (DLS) technique. 

*Determination of loading efficiency (LE%)*

The LE% of the NPs was calculated as the percentage ratio of the actual SIL methanolic extract loading of the particles to the primary SIL applied to the composite NPs. The entrapped amount of SIL in NPs (CH-SIL NPs) was calculated after centrifugation at 13000 rpm for 15 min to separate the unloaded SIL (supernatant) and SIL-loaded NPs (19). LE% was calculated with the following equation: LE% = (A - B)/A × 100

A = Total amount of primary SIL used to make the NPs and B = Total amount of SIL in the supernatant after centrifugation.

*X-ray diffractometer (XRD)*

X-ray diffraction techniques are used to identify crystalline phases of various materials and the quantitative phase analysis after the identification by D8-ADVANCE (Bruker, Germany).

*Scanning electron microscopy (SEM)*

The external morphology of particles is evaluated by observing a Scanning Electron Microscopy (SEM) using TESCAN-Vega 3 (TESCAN SEM, Czech Republic).

*Animals and study design*

Twenty-four male Wistar rats weighing 200 ± 30 g (Pasteur Institute of Iran) were housed in standard cages in a controlled environment (23 ± 2 °C, relative humidity 55 ± 5%, 12-hr light/dark cycle) and fed *ad libitum*. Animal conservation and experimental procedures were accepted by the Animal Lab Ethics Committee of the Science and Research Branch (approval ID IR.IAU.SRB.REC.1398.187) at the Islamic Azad University. Eighteen Wistar rats were fed a high-fat diet (HFD) with excessive cholesterol to induce the hyperlipidemic rat model (10). The HFD was made by mixing powdered commercial standard food (60.5%), lard (30%), cholesterol (4%), white sugar (5%), and cholic acid (0.5%) in the high-fat diet (4, 20) for twelve weeks. After a 1-week acclimatization period on a regular diet, rats were randomly assigned into four dietary groups (n = 6 for each group): a standard diet group (ND), the HFD group, the S+HFD group fed SIL (15 mg/kg) for twelve weeks and HFD, the CHS+HFD group ate silymarin-loaded chitosan nanoparticles (15 mg/kg) (18) and HFD for twelve weeks.Body weight and food consumption were measured weekly. SIL and CH-SIL NPs were dissolved in distilled water and administered orally by gavage (0.5 ml for each rat).

At the end of the treatment period (12 weeks), under deep anesthesia using 33% CO_2_/66% O_2_, blood was collected from the heart to separate serums to measure lipid profiles and hormone levels (testosterone, cortisol, and insulin) by centrifugation at 3000 g for 15 min at 4 °C. HOMA-IR was calculated as (fasting serum glucose × fasting serum insulin) / 22.5.The brain was dissected and stored at -80 °C to measure NPY, Y1R, and Y5R expression gene levels. RNA isolation and RT-qPCR procedures have been designated before (21).

*Determination of lipid profile*

The blood was allowed to stand at room temperature for 30 min, centrifuged at 3000 r/min for 15 min, and the supernatant was obtained. The contents of total cholesterol (TC), triglyceride (TG), and high-density lipoprotein cholesterol (HDL-C) in the serum were measured using kits according to the information provided with the kits. In addition, the LDL-C and VLDL of all groups were calculated. Serum TG and TC levels were measured by a biochemical analyzer using commercial kits (Olympus AU-600, Tokyo, Japan). HDL-C was analyzed by a Pars Azmoon kit from Iran. LDL and VLDL were calculated by Friedewald, LDL-C = TC - (HDL-C + TG/5), (VLDL-C = TG/2.22) (22). 

*Assessment of biochemical parameters in serum*

Serum levels of testosterone, cortisol, and insulin were measured by the rat enzyme-linked immunosorbent assay using commercial kits from Biotech CO (Shanghai, China) following the manufacturer’s information. Blood glucose was measured at the end of the last day of treatment using a glucose enzymatic kit (Megazyme, Ireland).Also, the amount of insulin resistance was determined based on the homeostasis model (23).

***Gene expression***

Total RNA was extracted from rat hypothalamic tissue with a special RNeasy Mini kit (Qiagen) according to the manufacturer’s instructions. The nanodrop device confirmed the quality of extracted RNA and cDNA was synthesized using the Revert Aid First Strand cDNA Synthesis Kit (Thermofisher). Quantitative RT-PCR was performed with an initial SYBR Premix Ex Taq II Kit (CinnaGen, Iran) on a thermocycler system(StepOneplus, Thermo Fisher Scientific, Germany) incubation at 95 °C for 30 sec, followed by 38 cycles of 15 s at 94 °C and 1 min at 62 °C. The relative expression of the target genes was obtained using the GAPDH housekeeping gene. The detection system software confirmed the threshold cycle one number, and data were analyzed using the 2^∆∆CT^ method (21). Each reaction was carried out in triplicate, and statistical analysis was done via SPSS version 25. The primers used for qRT-PCR are as follows: Forward 5-GCT AGG TAA CAA ACG AAT GGG G-3´ and reverse 5-CAC ATG GAA GGG TCT TCA AGC-3´ for NPY gene, forward 5-TCTCATCGCTGTGGAACGTC-3´ and reverse primers were CCGCCAGTACCCAAATGACA for Npy1R gene (24), forward 5-GCCGAAGCATAAGCTGTGGAT-3’ and reverse 5´-TTTTCTGGAACGGCTAGGTGC-3´ for Npy5R gene (21).

***Statistical analysis***

Data analysis was done with GraphPad Prism version 8.1 and SPSS version 25. After assessing the normal distribution, the one-way analysis of variance (ANOVA) test was used, followed by Tukey’s *post-hoc* test for multiple comparisons. Gene expression data complied with normality and equal variance expectations, complete with Shapiro-Wilk and Levene’s tests for equal variance. Variances between groups were assessed using an unpaired Student’s t-test. *P*<0.05 was statistically significant. Values are expressed as means ± standard deviations or standard error.

## Results

***Physicochemical characterization***

*Zeta potential, particle size, and loading efficiency (LE%)*

Zeta potential was achieved as +10.4 ± 1.3 mV. The mean diameter of SIL-loaded CH particles was 194.4 ± 40.2 nm, and the PDI was 0.508. The percentage of LE was achieved as 82.7% (Table 1). 

*Fourier transform infrared spectroscopy (FTIR)*

**Figure 1:**
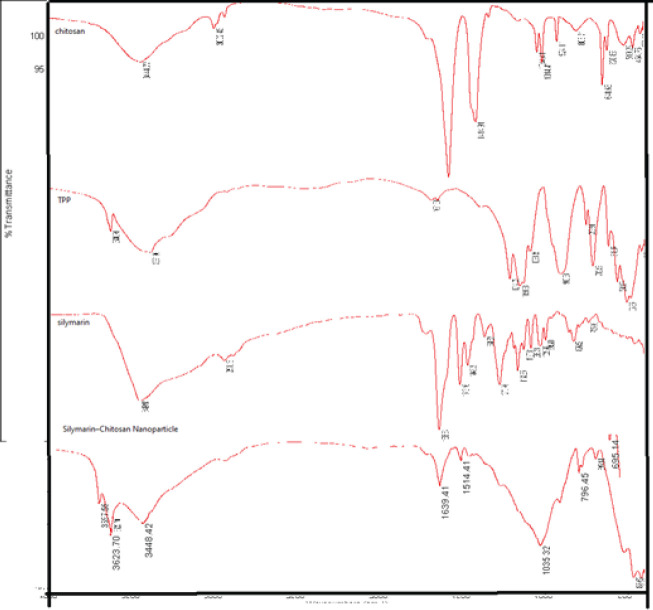
FTIR spectra of TPP, chitosan, silymarin, and silymarin-loaded chitosan nanoparticles

The FTIR spectra of CH, SIL, and SIL-loaded CH NPs are shown in Figure 1. The CH spectrum shows typical absorption peaks at 3365, 2915, and 2840 cm^-1^ related to -NH_2_, -CH_2_, and -CH_3_ stretching vibrations. Peaks around 1380 and 1418 cm^-1^ were attributed to the -CH3 and -OH vibrations, respectively. The peak around 1318 cm^-1^ was a C-N stretching vibration of type I amine. The combination vibration region, 1095 to 990 cm^-1^, is related to sugar rings in the CH structure. Finally, the C-N fingerprint peak emerged at 793 cm^-1^. One of the prominent peaks in the CH spectrum is 1588 cm^-1,^ related to the C=O stretching of type I amide. This peak somewhat shifted to 1538 cm^-1^ in the CH-SIL NPs. Furthermore, the CH-NPs exhibit -N-H stretching at 1035 cm^−1^. The peaks at wavenumbers 1590 and 1650 cm^-1^ shifted to 1580 and 1640 cm^-1^ related to the N-H bending vibration of amine І and the amide ІІ carbonyl stretch, respectively (Figure 1). A P=O peak at 1200 cm^-1^ in CH NPs can be associated with the linkage between phosphoric, and ammonium ions of TPP and ammonium groups of CH A -C-C=C asymmetric stretch at 1547 cm^−1^ is present in both SIL and SIL-loaded CH NPs. The SIL spectrum revealed peaks at 3000 cm^-1^ corresponding to C-H stretching vibration and at 2925 and 2853 cm^-1^ assigned to the asymmetric and symmetric stretching vibration of C-H methyl groups. The bands at 1458 and 1735 cm^-1^ refer to the CH_2_ groups bending vibration and C=O stretching vibration, respectively. The band at 1162 cm^-1 ^can be recognized as C-O ester group stretching vibrations, and 743 cm^-1^ corresponds to the CH_2_ bending vibration of chains with more than seven carbon atoms (18). The FT-IR spectrum of pure SIL and SIL-loaded CH NPs displayed an absorption band at 3447.22 cm^-1^ (O-H stretching, Phenols/Alcohols), 2938.18 cm^-1^ (C-H stretching, Alkyl), 1638.15 cm^-1^ (C=O stretching), 1513–1466.72 cm^-1^ (aromatic C=C ring stretching), 1363 cm^-1^ (N-O stretching), 1272 cm^-1^ (C-O stretching, Carboxyl acid), 1162 cm^-1^ (C-O stretching, Esters), 1084 cm^-1^ (benzopyran ring), 1029 cm^-1^ (C-O group stretching, Sulfoxide), 823 cm^-1^ (C-H bending, Alkenes), and 607 cm^-1^ (C-I stretching, halo compound). The spectrum of CH-SIL-NPs revealed all typical peaks and coordinated with individual vibrational bands of pure drug SIL with a slight shift in peak positions (14, 15, 18). 

*X-ray diffractometer (XRD)*

Lack of characteristic peaks of the drug in the XRD diffractogram of microbeads indicates entrapment of the drug within the polymeric matrix of microbeads with 34.5% crystal form (Table 1).

*Scanning electron microscopy (SEM)*

**Figure 2:**
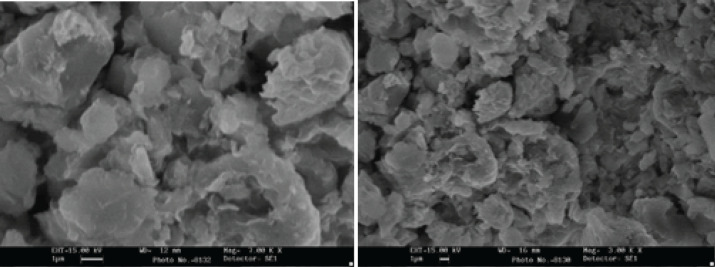
Scanning electron microscopy (SEM)of the chitosan-silymarin nanoparticle

Investigation of the CH-SIL NPs displayed that the average particle size of the CH-SIL NPs was 194.4 ± 40.2 nm in diameter. However, the SEM (20000x) investigation established that the particles were clustered and appeared as tiny beads in CH-SIL NP form (Figure 2). 

*Effect of CH-SIL NPs on body weight and food intake*

**Figure 3:**
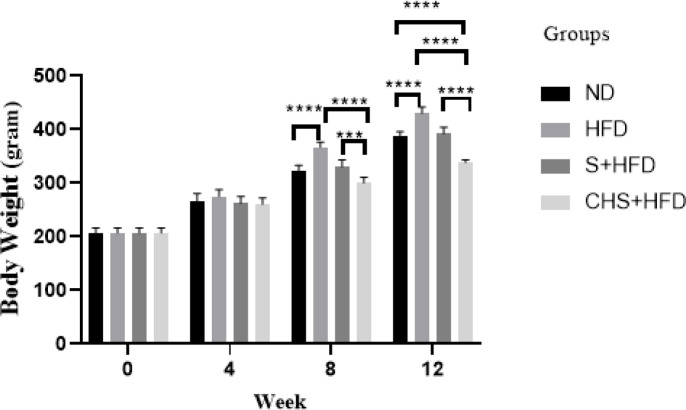
Effects of SIL-CH NPs on body weight in hyperlipidemic rats

**Figure 4:**
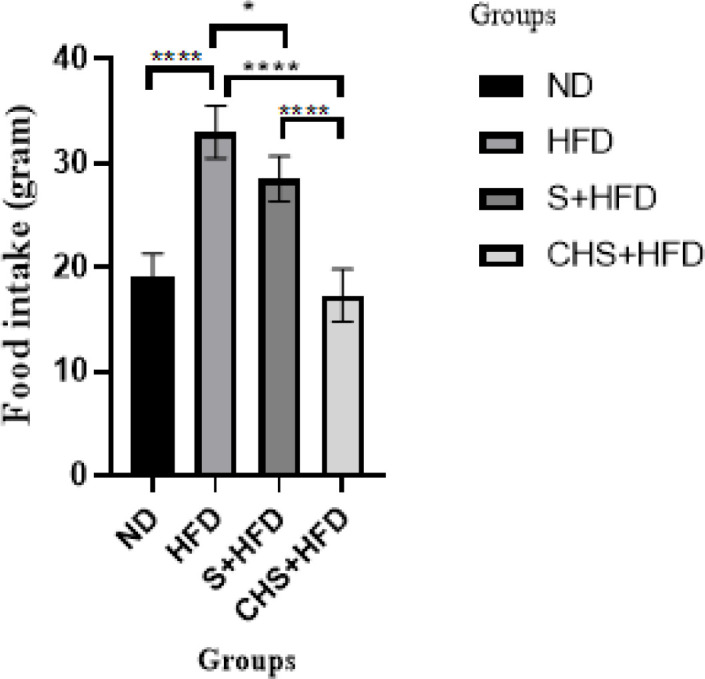
Effects of SIL-CH NPs on food intake in hyperlipidemic rat

The HFD group was 16.9% overweight after twelve weeks of receiving the high-fat diet compared to the healthy group, with a statistically significant difference (*P*<0.0001). 39.4% weight loss in the CHS+HFD group was observed, compared to the HFD group (*P*<0.0001). Significant weight loss was observed in the S+HFD (*P*<0.0001) and CHS+HFD (*P*=0.0001) treatment groups compared to the hyperlipidemic group. In addition, a significant difference (*P*<0.0001) was observed between S+HFD and CHS+HFD treatment groups, about 13.7% (Figure 3). A significant reduction in food intake was displayed in the CHS+HFD group compared to the HFD group (*P*<0.05). The CHS+HFD group showed a significant decrease compared to the S+HFD group (*P*<0.01), but no significant difference was observed between the healthy group and the CHS+HFD group (Figure 4).

*Effect of CH-SIL NPs on serum lipid profile in hyperlipidemic rats*

The serum lipid profiles of rats after twelve weeks of treatment were shown in Table 2 compared to those of the ND group receiving standard rodent chow, serum levels of TC and TG in the model group enhanced approximately by 113% and 55.6% (*P*<0.01), and HDL-C serum level decreased by 31.8% (*P*<0.0001). Oral administration of CH-SIL NP treatments resulted in a significant reduction of TC (*P*=0.0006) and TG (*P*=0.02) and elevation of HDL-C levels (*P*< 0.001) compared with the HFD group (Table 2). 

*Effects of silymarin-loaded chitosan nanoparticle (CH-SIL) on circulating glucose and hormone level*

A significant decrease was observed in the insulin and cortisol between HF and the other groups, including S+HFD (*P*=0.0092), CHS+HFD (*P*=0.0002), and ND groups (*P*=0.0001). The testosterone was significantly higher in the CHS+HFD group than in the other groups and was markedly lower in the HFD group than in the CHS+HFD (*P*=0.0386) and S+HFD(*P*=0.004) groups. Nevertheless, no significant difference was detected between S+HFD vs CHS+HFD. Consumption of HFD for twelve weeks enhanced blood glucose (277.0 ± 20.14 vs 147.9 ± 18.06, *P*=0.0028) as indicated by higher HOMA-IR (3.59 ± 0.03 vs 1.41± 0.14, *P*=0.0014) compared with healthy rats. As revealed in Table 2, CH-SIL NP administration for twelve weeks to hyperlipidemic rats decreased the concentration of serum glucose by 19.6% (*P*=0.038) and serum insulin by 31.8% (*P*=0.0075) and increased testosterone by 71.61% (*P*<0.001). In addition, CH-SIL NPs administration to the rats decreased HOMA-IR by 97.3% (*P*=0.0058) and cortisol by 50% (*P*=0.023, Table 2).

*Effect of CH-SIL NPS on mRNA expression of NPY, Y1R, and Y5R*

**Figure 5:**
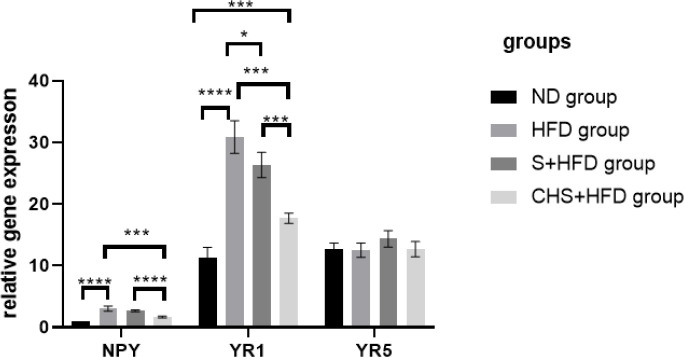
Effects of SIL-CH NPs on mRNA expression of NPY, Y1R, and Y5R levels. ND group: healthy rats (n = 6); HFD group: High-fat diet; S+HFD group: silymarin (15 mg/kg) + high-fat diet, CHS+HFD, CHS group, silymarin-loaded chitosan nanoparticles (15 mg/kg) +high-fat diet; * *P*<0.05, ** *P*<0.01, *** *P*<0.001, **** *P*<0.0001. Values are mean ± SEM

In rats, twelve weeks of HFD consumption induced hyperlipidemia by significantly increasing orexigenic NPY by 202% (*P*=0.0003) and concomitantly enhancing Y1 receptor expression by 172.3% (*P*<0.0001), as compared with the ND group. Y5R mRNA levels were unaffected (Figure 5). Administration of CH-SIL NPS inhibited NPY mRNA expression by 44.6% (*P*=0.0009) and Y1R mRNA level by 42.7% (*P*<0.0001)induced by HFD in hyperlipidemic rats. The NPY (*P*<0.0001) and Y1R (*P*<0.0002) were markedly lower in the CHS+HFD group than in the S+HFD group (Figure 5).

***Correlation studies***

The decrease in body weight, food intake, insulin, and cortisol was associated with variations in the gene expression of neuropeptides and Y1 receptors. However, the increase in testosterone was related to changes in NPY and Y1R mRNA expression levels. 

**Table 1:** Zeta potential and particle size of silymarin-loaded chitosan nanoparticles (CH-SIL) (n = 6)

Formulation	Particle size ‎(nm)	Zeta potential ‎(19)	Loading efficiency ‎(%)‎	Polydispersity index ‎	XRD crystal%
CH-SIL	‎194.4 ± 40.2	+10.4 ± 1.3	82.7	0.508 ± 0.002	34.50

ND group: healthy rats (n = 6); HFD group: High-fat diet; S+HFD group: silymarin (15 mg/kg) + high-fat diet; CHS+HFD, CHS group: silymarin-loaded chitosan nanoparticles (15 mg/kg) + high-fat diet; * *P*<0.05, ** *P*<0.01, *** *P*<0.001, ****P<0.0001. Values are mean ± SEM

ND group: healthy rats (n = 6); HFD group: High-fat diet; S+HFD group: silymarin (15 mg/kg) + high-fat diet; CHS+HFD, CHS group, silymarin-loaded chitosan nanoparticles (15 mg/kg) + high-fat diet; * *P*<0.05, ** *P*<0.01, *** *P*<0.001, ****P<0.0001. Values are mean ± SEM

**Table 2:** Effect of CH-SIL NPs on serum lipid profile, insulin, HOMA-IR, and glucose in hyperlipidemic rat

**Parameter**	**ND**	**HFD**	**S+HFD**	**CHS+HFD**	***P*** **-value**
**TG ** **(mmol/L)**	0.26 ± ‎0.01	0.58 ± ‎0.03^***^	0.45 ± ‎0.01^**^	0.39 ± ‎0.01^**a^	*P*<0.0001
**TC ** **(mmol/L)**	2.21 ± ‎0.06	2.7 ± 0.05^**^	2.1 ± 0.03^b^	2.058 ± 0.04^*c^	*P*<0.0001
**HDL-C ** **(mmol/L)**	1.2 ± ‎0.05	0.62 ± 0.03^**^	0.57 ± 0.2^**^	0.72 ± 0.03^**^	*P*=0.0093
**LDL-C ** **(mmol/L)**	1.07 ± 0.05	1.52 ± 0.04^**^	1.16 ± 0.3^b^	0.97 ± 0.04^bA^	*P*<0.0001
**Glucose (mg/dl)**	147.9 ± 18.06	277.0 ± 20.14^**^	244.0 ± 36.14^**^	222.7 ± 15.76^***a^	*P*=0.0268
**Insulin (ng/dl)**	21.38 ± 0.62	29.17 ± 0.88^***^	23.13 ± 0.84^b^	19.89 ± 0.74^b^	*P*=0.0002
**HOMA-IR**	1.41± ‎0.14	3.59 ± 0.03^**^	2.65 ± 0.03^**a^	1.97 ± 0.03^*b^	*P*<0.0001

ND group: healthy, HFD group: High-fat diet; S+HFD group: silymarin (15 mg/kg + high-fat diet); 4-CHS+HFD group, CHS, silymarin-loaded chitosan nanoparticles (15 mg/kg + high-fat diet), ND vs HFD, S+HFD, and CHS+HFD: * *P*<0.05, **P<0.01, ***P<0.001. HFD vs S+HFD and CHS+HFD: a *P*<0.05, b *P*<0.01, c *P*<0.001. S+HFD vs CHS+HFD: A *P*<0.05; Values are mean ± SEM

## Discussion

This work aims to prepare silymarin (SIL)-loaded chitosan (CH) nanoparticles (SIL-CH NPs) as a new weight loss agent, which was formulated by loading the SIL particle on chitosan NPs to expand anti-dyslipidemia effects and improve hypothalamic NPY level of SIL in a rat model of diet-induced hyperlipidemia. In previous studies, SIL has been administered in higher doses because of poor bioavailability in the brain (13). CH, a novel drug delivery system, could efficiently enhance oral bioavailability (25). DLS (Table 1), SEM (Figure 2), and FTIR (Figure 1) investigations proved the successful synthesis of SIL-loaded CH NPs. DLS and SEM images displayed a narrow size dispersal of around 170–230 nm with an almost spherical shape, affecting absorption and biodistribution (15). The design of these carriers was based on natural chitosan polymers to promote oral drug bioavailability and intestinal drug absorption of SIL. It was consistent with earlier available SIL-CH NP principles (18). Chitosan has less affinity for water, forming a slow-release drug from the formulation (26). A study on chitosan hydrogels determined that when the concentration of chitosan increases, the release of the drug becomes slower (14). The other findings revealed that chitosan could be used as an inexpensive and nontoxic technique that is safe for the environment and drug delivery system compared with synthetic compounds for improving the bioavailability and biodegradation of drug yield production (26, 27). The size of NPs influences the permeability of the particle across the blood-brain barrier, and adding chitosan as a carrier leads to an active substance for sustained release in the intestinal tract (28).

In this study, our results displayed that oral administration of a high-fat diet for twelve weeks caused the development of dyslipidemia, hormonal disorders, and weight gain, confirmed by higher food consumption, HOMA-IR, cortisol, NPY mRNA expression levels, and testosterone degradation compared with healthy rats. Administration of a high-fat diet with excessive cholesterol for eight weeks in male Wistar rats resulted in diet-induced obesity (DIO), atherosclerotic model (29), and hypercholesteremia (22, 30). HDL-C attains a vital function of declining cholesterol levels in the peripheral tissues and blood and prevents atherosclerosis plaque development in the aorta (31). Increased serum TG levels were consistent with amplified rate of coronary artery disease (29). This study revealed that the HFD group demonstrated significantly high body weight. These results align with the findings of earlier studies and recommend that CH-SIL NPs could inhibit the development of hyperlipidemic rat models. SIL-CH NP treatment has been shown to reduce body weight, serum cortisol, TG, cholesterol, LDL, and hypothalamic NPY levels; however, it increased HDL and testosterone in hyperlipidemic rats against HFD-induced damage. 

In the present study, HFD-induced hyperlipidemia was correlated with a significant increase in cortisol, insulin, NPY, and Y1 mRNA levels. Thus, suppressing hyperlipidemia, Y1R, and cortisol is a practical approach to protecting against obesity and metabolic disorders (32). Red ginseng is a supplementary medicine for physical and mental voiding, and baclofen is a muscle relaxant and antispasmodic medication. Both are potential new antiobesity drugs by attenuating appetite via reducing NPY expression (2, 33). The treatment of ginsenosides controlled the body weight, fat mass, and overexpression of hypothalamic NPY by activating AMP-activated protein kinase (AMPK), an intracellular sensor for lipid metabolism in obese rats (34). Flaxseed polysaccharide revealed antiobesity and hypoglycemic effects via down-regulating the hypothalamic NPY and activating the AMPK signaling pathway to enhance lipolysis and fatty acid oxidation (35). Anthocyanins, secondary metabolites of flavonoids, were correlated with reduced body weight and food intake via modulation of NPY and GABA receptors in the hypothalamus (36).

The most appropriate finding of this study is that the activity of silymarin-loaded chitosan nanoparticles as a mucoadhesive carrier enhances the correlation positively with HDL and testosterone and negatively with cortisol and TG in treated hyperlipidemic male rats. In addition, the effect of CH-SIL NPs on body weight and food intake agreed with other reports showing a slightly reduced body weight. This slight but significant decrease in the average body weight of the CHS+HFD group may be caused by the mucoadhesive property of chitosan, which increases drug absorption and half-life compared to rats receiving SIL. Administration of SIL (50 mg/kg) may progress the fatty acid β-oxidation and gluconeogenesis to reduce triglyceride serum levels and improve blood glucose through expression of pyruvate dehydrogenase kinase 4 and AMPK knockdown and subsequently improve glucose and lipid metabolism in exercise(37). SIL could diminish serum triglyceride and blood sugar levels, and LDL levels differed significantly between S+HFD and CHS+HFD groups. By measuring glucose and insulin levels, SIL can improve insulin resistance (38). Our result of diminished HOMA-IR levels in the CHS+HFD group is consistent with the previous study. These effects may decrease serum glucose and insulin. It has been demonstrated that changes in lipid concentrations and lipoprotein fractions are related to increased risk for obesity-related metabolic conditions and development of dyslipidemia (38). SIL reduced cholesterol absorption in rats fed a high-cholesterol diet. It caused significant decreases in liver VLDL, cholesterol, and TG and could contribute to the positive changes in plasma cholesterol lipoprotein profile (39). These data,together with those of serum insulin, clearly show that CH-SIL NPs could partially inhibit some effects of HFD-induced overweight. CH-SIL NP treatment may reverse the results of a high-fat diet on body weight, and this effect may be related to decreased fat absorption. 

Treatment of SIL for eleven weeks (200 mg/kg/day) significantly reduced body weight and food intake in HFD-induced obese rats.Previous research has stated that administering *S. marianum* seeds-infused solution (200 mg/kg/day) for six weeks (40) and SIL (150 mg/kg) (33) in rats boost plasma testosterone levels significantly as an aromatase inhibitor, blocking the alteration of androgens to estrogens. Meanwhile, silybin may stimulate Leydig cells to produce testosterone.These results agree with earlier research indicating the advantage of using *S. marianum* extract, SIL, or silybin to increase testosterone levels. However, a very low dose of SIL was used in our research. SIL exhibited antidepressant-like activity, reduced serum corticosterone levels, MDA formation, restoration of anti-oxidant enzymes, SOD, and CAT in the hippocampus and cerebral cortex in the model of acute restraint stress (41). Infrared spectroscopy was done to evaluate the formation of CH-SIL nanoparticles and inspect the likely interactions between SIL and other compounds. This finding can approve the SIL loading in CH of its prominent peaks on the spectrum of SIL-loaded CH NPs. Furthermore, the surface charge of the particles as zeta potential for CH-SIL NPs was obtained as +10.9 ± 5 mV, which was appropriate to adhere to the negatively charged intestinal mucus layer, as the higher the zeta potential, the better the adhesion to the mucus (15, 18). Therefore, the final charge on the particle will be more positive, which is proper for adhering to the mucosa.

The SEM investigation of the CH-SIL NPs indicated that the average particle size of CH-SIL NPs was 205 ± 42 nm in diameter. Particles with a size between 100 and 1000 nm are appropriate to the micro-nano grade particles. SEM revealed that the particle size of the DLS was greater than that of the SEM. Therefore, it could be implicit that nanoparticle size was reduced due to the loss of humidity content from the samples and the nanoparticles getting closer to each other.Furthermore, it was demonstrated that the chitosan nanoparticle was formed with poor dispersion and had spherical clusters with accumulation.A previous study revealed that chitosan NPs established a suitable mucoadhesive property directly correlated with the mucoadhesive polymer concentration (26). When an anorexigenic signal is stimulated, food intake diminishes.

Pretreatment with CH-SIL NPs reduced the lipid peroxidation reactions and enhanced the activities of SOD, CAT, and GPx, in cerebral ischemia/reperfusion rats. Also, CH-SIL NP pretreatment produced a significant decrease in the expression of IL-6 and TNF-α in the cortex compared to SIL pretreatment (18).

Administration of the CH-SIL NPs to hyperlipidemic male rats significantly decreased body weight and food consumption compared with the S+HFD and ND group (*P*<0.01), suggesting the weight loss effect of silymarin-loaded chitosan. It may indicate that the silymarin-loaded chitosan at 15 mg/kg/day has a better weight loss effect than SIL at 15 mg/kg/day in this animal model. Hypercholesterolemia is related to the pathogenesis of obesity (29). Meanwhile, TC, TG, and LDL levels were significantly decreased; HDL-C levels increased in the CHS+HFD group compared to the HFD group, suggesting a hypocholesterolemic property of silymarin-loaded chitosan. SIL modulated the visceral adipose tissue’s intracellular lipid flow (fatty acid transport and oxidation) (39).

## Conclusion

The results showed that oral administration of a high-fat diet for twelve weeks caused the development of dyslipidemia, hormonal disorders, and weight gain. CH-SIL NPs could inhibit the development of hyperlipidemic rat models and reduce body weight, serum glucose, cortisol, TG, cholesterol, LDL, and hypothalamic NPY levels. Suppressing hyperlipidemia, Y1R expression level and cortisol by administration of CH-SIL NPs is a practical approach to protecting against weight gain and metabolic disorders. 

## Authors’ Contributions

N P, F M, S S, and G A designed the experiments; VA performed the experiments and collected data; FM, S S, and G A discussed the results and strategy; N P supervised, directed, and managed the study; N P, F M, S S, and G A approved the final version to be published.

## Funding

Funding This research received no specific funding.

## Ethics Approval

This study was approved by the National Committee on Ethics in Biomedical Research (ethics.research.ac.ir) of the Islamic Azad University, Science and Research Branch and(approval ID IR.IAU.SRB.REC.1398.187).Moreover, according to Helsinki declaration, it was conducted with appropriate caution to respect the animals’ welfare in this research.

## Conflicts of Interest

The authors declare no conflicts of interest.
